# p53 nuclear accumulation as an early indicator of lethal prostate cancer

**DOI:** 10.1038/s41416-019-0549-8

**Published:** 2019-08-14

**Authors:** David I. Quinn, Phillip D. Stricker, James G. Kench, Judith Grogan, Anne-Maree Haynes, Susan M. Henshall, John J. Grygiel, Warick Delprado, Jennifer J. Turner, Lisa G. Horvath, Kate L. Mahon

**Affiliations:** 1 Norris Comprehensive Cancer Centre, University of Southern California, Los Angeles, USA; 2 St Vincents Prostate Cancer Centre, Sydney, Australia; 3 Garvan Institute of Medical Research, Sydney, Australia; 4 Royal Prince Alfred Hospital, Sydney, Australia; 5 University of Sydney, Sydney, Australia; 6 Union for International Cancer Control, Geneva, Switzerland; 7 St Vincents Hospital, Sydney, Australia; 8 Douglass Hanly Moir Pathology, Sydney, Australia; 9 Chris O’Brien Lifehouse, Sydney, Australia

**Keywords:** Prostate cancer, Tumour biomarkers

## Abstract

**Background:**

After radical prostatectomy (RP) for prostate cancer (PC), p53 alterations predict biochemical relapse (BCR), however, recent evidence suggests that metastatic relapse (MR) not BCR is a surrogate for PC specific mortality (PCSM). This updated analysis of a previously published study investigated the association between p53 aberrations, MR and PCSM in men with localised PC.

**Methods:**

Two hundred and seventy-one men with localised PC treated with RP were included. RP specimens stained for p53 by immunohistochemistry were scored as (a) percentage of p53-positive tumour nuclei; and (b) clustering, where ≥12 p53-positive cells within a ×200 power field was deemed ‘cluster positive’. Associations between p53 status and clinical outcomes (BCR, MR and PCSM) were evaluated.

**Results:**

Increasing percentage of p53-positive nuclei was significantly associated with shorter time to BCR, MR and PCSM (All *p* < 0.001). Half of the patients were p53 cluster positive. p53 cluster positivity was significantly associated with poorer outcomes at all clinical endpoints (BCR: HR 2.0, 95% CI 1.51–2.65, *p* < 0.001; MR: HR 4.1, 95% CI 2.02–8.14, *p* < 0.001; PCSM: HR 12.2, 95% CI 1.6–93; *p* = 0.016). These associations were independent of other established prognostic variables.

**Conclusions:**

p53 aberrations in radical prostatectomy tissue predict clinically relevant endpoints of MR and PCSM.

## Background

Prostate cancer (PC) is the most commonly diagnosed cancer and the second leading cause of cancer death in men in developed countries.^1^ Localised treatment is beneficial for a subset of patients but there is clear evidence of overtreatment in others. Understanding the fundamental biology is essential to identify better therapeutic targets while tools to optimally select those patients with potentially lethal disease at the outset will improve outcomes and avoid unnecessary morbidity.

p53 is a potent tumour suppressor, and mutation of the encoding *TP53* gene is a central element in the initiation and progression in at least half of all human cancers.^2,3^ TP53 mutations often result in nuclear accumulation of mutant p53 proteins, making it amenable to immunohistochemical analysis across multiple malignancies including PC.^4–6^ Somatic *TP53* mutations are found in up to 20% of patients with localised PC and are enriched in metastatic castration resistant PC tissue (53%), suggesting they may be a harbinger for aggressive disease.^7^ In patients receiving radiation and androgen deprivation therapy for locally advanced disease, p53 mutations are associated with poor prognosis, but the effect on response to specific treatments is contradictory across the two major studies.^8,9^

There is increasing evidence that metastatic relapse (MR) is a more accurate surrogate for PC specific survival than biochemical relapse (BCR).^10^ Recent work from our own group has shown that markers clearly associated with biochemical recurrence do not necessarily translate into markers of MR with more mature follow-up.^11^ In this cohort, p53 nuclear accumulation correlates with biochemical recurrence after radical prostatectomy (RP) as described by our group more than 15 years ago.^12^ With longer follow-up, we aimed to examine whether p53 could also predict MR and PC specific survival.

## Methods

### Study population

This retrospective study consisted of 271 men with localised PC treated with radical prostatectomy between 1988 and 1996 in a single institution (St Vincent’s Hospital, Sydney, Australia). The 271 cases for the study were selected from 409 RPs treated in the study period and were chosen due to the availability of archival tissue. They were not stratified for known pre-operative prognostic factors. A detailed description of the patient cohort has been previously reported.^12^ The study was approved by the St Vincent’s Research Ethics Committee (12/231).

### Pathological examination

Formalin fixed paraffin embedded (FFPE) radical prostatectomy specimens were examined contemporaneously for involvement of surgical margins, extraprostatic extension, seminal vesicle involvement, lymph node involvement, pTNM stage (AJCC 2010) and original (1992) Gleason score (GS).^12^ These details were extracted from the Australian Prostate Cancer Research Centre-NSW (APCRC-NSW) bio-resource and database. Histopathology sections were re-graded as per the 2005 ISUP modification of the Gleason grading system by three uropathologists blinded to the original GS and patient outcomes. All cases were assigned a GS and a 2014 ISUP grade (also known as grade group) in line with the recommendations of the 2014 ISUP consensus conference.^13^

### Immunohistochemical analysis

As described previously,^14^ immunohistochemistry was performed on formalin-fixed paraffin-embedded blocks sectioned at 5 microns, mounted on SuperFrost Plus slides (Menzel-Glaser, Germany) and processed within 10 days of sectioning. The mouse monoclonal p53 DO-7 epitope directed antibody (DAKO Corporation, Carpinteria, CA) and avidin-biotin-peroxidase and diaminobenzidine kits (Vector Laboratories, Burlingham, CA) were used according to manufacturer’s instructions. Sections were deparaffinised in xylene and rehydrated through graded ethanol then heated in a pressure cooker in 0.01 M citrate buffer at pH 6.0 for 10 min to enhance antigen retrieval. The sodium citrate buffer was brought to the boil in an open pressure cooker. The slides were placed in boiling buffer, the lid locked, and the pressure cooker was allowed to boil for 4 min before the hot plate was turned off. The pressure cooker was then allowed to cool for 10 min under gently running cool water. When pressure in the pressure cooker fell the lid was removed. The sections were then treated with 2% H_2_O_2_ for 10 min at RT to inactivate endogenous peroxidase activity. Following a blocking step with 10% normal horse serum, the sections were incubated with DO-7 antibody diluted to 1:200 in 2% BSA/PBS overnight at 4 °C. Subsequently, sections were sequentially incubated with a biotinylated horse antimouse IgG, avidin-biotinylated complex and diaminobenzidine. Counterstaining was undertaken with Whitlock’s haematoxylin and ‘light green’ before dehydration through graded ethanol and xylene and cover slipping. A contiguous section was stained with haematoxylin and eosin. Positive controls for p53 nuclear accumulation used with each run of staining were a paraffin embedded pellet of the prostate cancer cell line DU145,^14^ which has a documented *p53* mutation, a colon cancer specimen with *p53* missense mutation and a tongue cancer specimen with p53 nuclear accumulation. Negative controls used were a paraffin embedded pellet of the prostate cancer cell line PC3,^14^ which does not express p53 protein and the above described positive controls processed with the substitution of a non-immune mouse monoclonal antibody for the DO-7 antibody.

Scoring for p53 nuclear accumulation required assessment of all cancer in selected sections from an individual patient as described previously.^14^ Counting of a minimum of 200 cancer cells in each cancer (mean 812, range 210–2000 cells, median 607), was undertaken to determine the percentage of nuclei showing accumulation across all areas of cancer present. The target cell count was 500 per case were possible, but cancers with fewer cells than this were not excluded because of the potential for selection bias. Where the cancers had multiple foci, were extensive or heterogeneous, more cells were counted and selected from areas of varying p53 nuclear accumulation to provide a sample representative of staining across the entirety of the cancer. Additionally, assessors scored the cancers cluster positive if 12 or more cancer cell nuclei within any ×200 power microscopic field showed p53 accumulation. On arriving at the cluster definition, we relied initially on other workers’ observations using clusters of 15 cells^15,16^ and tested a variety of thresholds for the definition of a cluster within a range of 6–20 cells in the p53 score stratum with >0–2% p53 nuclear positivity (see below). Below 10 cells per ×200 field, there was no correlation with prognosis and positive cells tended to be dispersed throughout the cancer rather than be clustered within the area of a single field, whereas there were few cases with clusters of more than 15 cells within the >0–2% p53 score stratum. This definition differentiated for outcome when between 10 and 15 cells (log-rank *p* = 0.04 and *p* = 0.05, respectively, within the >0–2% stratum, whereas use of nine cells produced *p* = 0.12, and 16 cells produced *p* = 0.24) were included as thresholds and therefore 12 cells (log-rank *p* < 0.001) within the field was selected as a mid-point in this range. Sections were scored independently for p53 by two assessors (D.I.Q., S.M.H.) and one pathologist (W.D., J.J.T.), all of whom were blinded to patient outcome. The inter-observer Spearman rank coefficients for p53 score were between 0.92 and 0.96 signifying close agreement between scorers. Chi square test for the p53 cluster status initially assigned by different scorers produced *p* values of 0.87 and 0.76. Specifically, the two assessors identified 12 p53 cluster-positive cases not identified on initial assessment by the pathologist, while the pathologist identified two cases that were not initially identified by the assessors. All of these cases were deemed cluster positive at consensus review. Representative photomicrographs demonstrating p53 nuclear accumulation in PC are presented in Supplementary Fig. 1.

### Statistical analysis

Relapse was defined by the following criteria: BCR with a serum PSA concentration ≥0.2 ng/ml increasing over a 3-month period; MR as determined by a positive imaging scan confirming bony or visceral metastasis; PC-specific mortality obtained from the New South Wales State Cancer Registry and the patients’ general practitioners. Time to BCR, MR and PC-specific mortality were defined as interval between date of radical prostatectomy and the event.

Survival analyses were performed by Kaplan–Meier method. Associations between p53 score, p53 cluster status, established clinicopathological variables (pre-operative PSA, pathological stage, ISUP grade group, seminal vesicle involvement, extra-prostatic extension) and clinical outcomes (BCR, MR and PCSM) were measured using Cox proportional hazards models. Only those baseline variables that were significant on univariable analysis were included in multivariable analyses. Mann–Whitney U analysis was performed for all correlations. All reported *p*-values were two-sided. Statistical analyses were performed using SPSS version 24 (IBM, Armonk, NY, USA).

## Results

Clinicopathological characteristics for the 271 men included in the study are detailed in Table 1. At a median follow-up of 15.8 years (range 4 months–24.3 years), 203 patients (75%) had relapsed: 156 patients (57.5%) had a biochemical only relapse, 42 (15%) patients had experienced a metastatic relapse and 25 (9%) patients had died from PC. The all-cause mortality was 45.3%. The median time to BCR was 1.8 years, compared to MR (4.7 years) and PC-specific death (9.7 years).

**Table 1:** Clinicopathological characteristics (*n* = 271)

Characteristic	Number (% or range)
Age at RP (years)	64 (43–76)
Length of follow-up (years)	15 (0.3–24)
Pre-operative PSA (ng/mL)	11 (1–280)
Pathological T stage	
T2N0	127 (47)
T3aN0	87 (27)
T3bN0	45 (17)
T4N0	5 (2)
TxN+	6 (2)
Updated Gleason grade (ISUP grade group)	
≤6 (1)	49 (20)
3+4 (2)	108 (45)
4+3 (3)	47 (12)
8 (4)	12 (5)
≥9 (5)	25 (10)
Extraprostatic extension	138 (51)
Seminal vesicle invasion	50 (18)
Margin involvement	140 (52)
Adjuvant therapy	
Radiotherapy	12 (4)
Androgen deprivation therapy	39 (14)
Radiotherapy and androgen deprivation therapy	5 (2)
Clinical outcome	
Biochemical relapse	156 (58)
Metastatic relapse	42 (15)
Prostate cancer death	25 (9)
Death from any cause	123 (45)

### Percentage of p53-positive nuclei

p53-positive tumour nuclei were found in 210/271 (78%) patients. Increasing percentage of p53-positive nuclei correlated significantly with higher pathological stage (*p* < 0.001), ISUP grade group score (*p* = 0.001) and pre-operative PSA levels (*p* < 0.001) (Supplementary Table 1).

**Fig. 1:**
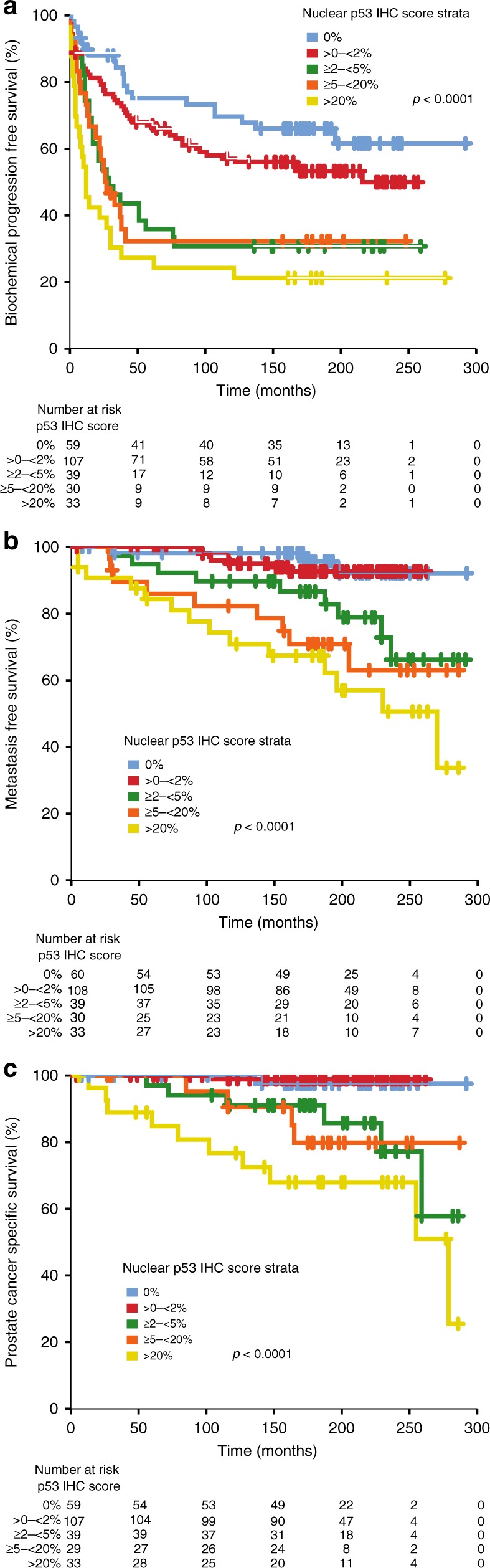
Kaplan–Meier analysis of nuclear p53 score strata for **a** biochemical progression-free survival, **b** metastasis-free survival, **c** prostate cancer specific survival

Increasing percentage of p53-positive nuclei was significantly associated with shorter time to BCR (HR 1.3, 95% CI 1.2–1.5; *p* < 0.0001, Fig. 1a), metastatic relapse (HR 1.9, 95% CI 1.5–2.3; *p* < 0.0001, Fig. 1b) and PC-specific mortality (HR 2.4, 95% CI 1.7–3.3; *p* < 0.0001, Fig. 1c).

### p53 cluster status

Half of all the patients were p53 ‘cluster positive’. All patients with >2% p53-positive nuclei were also ‘cluster positive’.

p53 cluster positivity correlated significantly with higher pathological stage (*p* < 0.001), Gleason grade (*p* = 0.001) and baseline PSA levels (*p* < 0.001) (Table 2).

**Table 2:** Multivariable analysis of p53 nuclear staining and established baseline prognostic variables and their association with biochemical relapse, clinical relapse and prostate cancer-specific survival

	% of p53-positive nuclei model HR (95% CI), *p*-value			p53 cluster model HR (95% CI), *p*-value		
Variable	*BCR*	*MR*	*PCSM*	*BCR*	*MR*	*PCSM*
Increasing p53 positive tumour nuclei^a^	1.2 (1.1–1.3) *p* = 0.006	1.4 (1.1–1.8) *p* = 0.007	1.9 (1.2–2.8) *p* = 0.003			
p53 cluster Positive vs negative				1.7 (1.2–2.4) *p* = 0.002	3.1 (1.1–8.6) *p* = 0.03	9.4 (1.2–76) *p* = 0.04
Lymph node involvement^b^ Present vs absent	*p* = 0.6	8.3 (1.8–38) *p* = 0.006	*p* = 0.9	*p* = 0.3	12.4 (2.8–54) *p* = 0.001	*p* = 0.8
ISUP grade group						
1	1	1	1	1	1	1
2	1.7 (1.0–2.8) *p* = 0.04	*p* = 0.9	*p* = 0.8	1.7 (1.0–2.7) *p* = 0.04	*p* = 0.9	*p* = 0.8
3	2.2 (1.2–3.8) *p* = 0.006	*p* = 0.2	*p* = 0.6	2.1 (1.2–3.6) *p* = 0.01	*p* = 0.2	*p* = 0.4
4	*p* = 0.07	*p* = 0.1	*p* = 0.9	2.3 (1.1–5.1) *p* = 0.04	*p* = 0.1	*p* = 0.9
5	*p* = 0.1	6.3 (1.2–32) *p* = 0.03	*p* = 0.2	*p* = 0.06	7.3 (1.4–37) *p* = 0.02	*p* = 0.1
Baseline PSA^c^, ng/ml	*p* = 0.6	*p* = 0.08	*p* = 0.8	*p* = 0.6	*p* = 0.7	*p* = 0.7

*HR* hazard ratio, *CI* confidence interval, *BCR* biochemical relapse, *MR* metastatic relapse, *PCSM* prostate cancer specific mortality, *ISUP* International Society of Urological Pathology

^a^Continuous variable

^b^Lymph nodes involved in 6/271 (2%) patients

^c^Continuous variable, log transformed

**Fig. 2:**
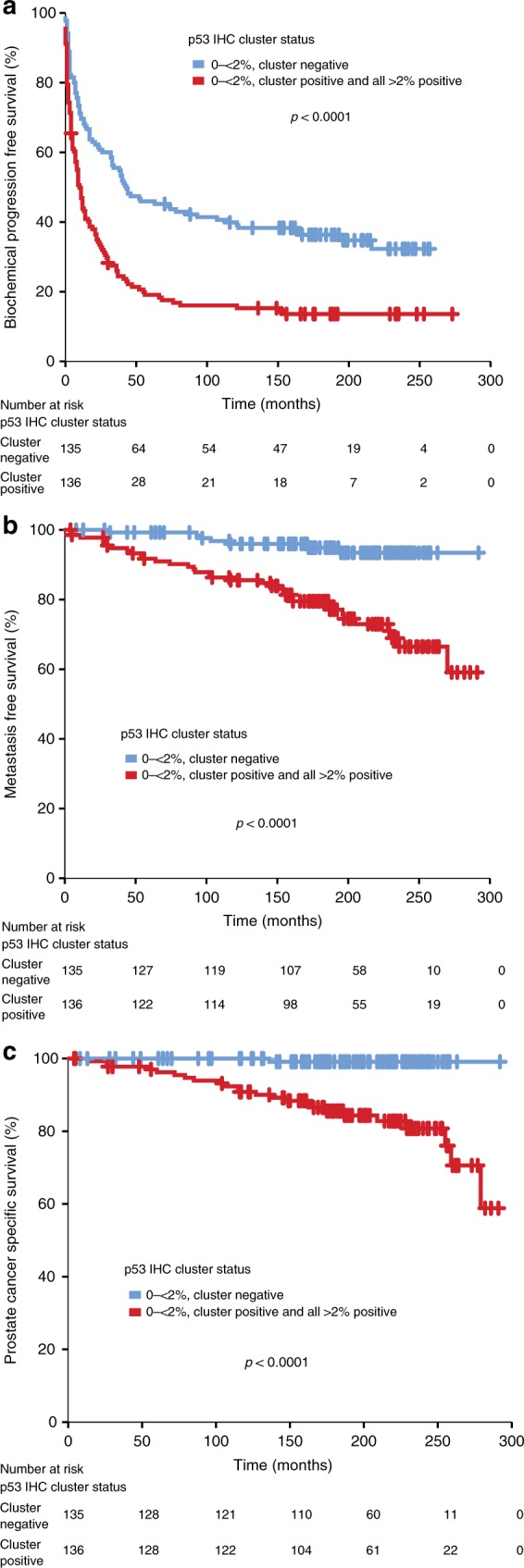
Kaplan–Meier analysis of nuclear p53 cluster status for **a** biochemical progression-free survival, **b** metastasis-free survival, **c** prostate cancer specific survival

p53 cluster positivity was also significantly associated with shorter time to BCR (HR 2.0, 95% CI 1.5–2.7; *p* < 0.0001, Fig. 2a), metastatic relapse (HR 5.1, 95% CI 2.3–11.5; *p* < 0.0001, Fig. 2b) and PC-specific mortality (HR 21.8, 95% CI 2.9–162; *p* = 0.003, Fig. 2c). Remarkably, only 1/135 (<1%) patients who were p53 cluster negative died from PC over the follow-up period.

On multivariable analyses, including established prognostic variables (lymph node status, pre-operative PSA and ISUP grade group score), p53 nuclear accumulation was independently associated with all clinical outcomes including MR and PC-specific mortality (Table 2 and Supplementary Table 2). The association with p53 was evident whether it was measured by increasing percentage of p53-positive nuclei as a continuous variable (MR, HR 1.4, 95% CI 1.1–1.8; *p* = 0.007; PC-specific mortality, HR 1.9, 95% CI 1.2–2.8; *p* = 0.003), by p53 cluster positivity (MR, HR 3.1, 95% CI 1.1–8.6; *p* = 0.03; PC-specific mortality, HR 9.4, 95% CI 1.2–76; *p* = 0.04), or using a dichotomous cut point of 10% or more (MR, HR 2.4, 95%CI 1.0–5.4; *p* = 0.04; PC-specific mortality, HR 4.9, 95% CI 1.7–15; *p* = 0.004).

## Discussion

This study, with extended follow-up, has clearly shown that altered p53 status as assessed by immunohistochemistry is significantly associated with the clinically relevant outcomes of MR and PC-specific death.

Clearly, p53 mutations are present early in the disease course, but impact the natural history of the disease many years later. The increased prevalence of p53 mutations in metastatic castration-resistant PC^7^ may be due to the early appearance of p53 mutations in subsequently lethal localised PC. While p53 has been long recognised and its role as a tumour suppressor well described,^17^ efforts to restore its tumour suppressing function have been so far unsuccessful.^3^ This study shows that there is still an argument for continuing to pursue drugs to successfully modulate this pathway as it appears to have a profound role in PC outcome. Neoadjuvant drug trials, prior to radical prostatectomy, are a well-described platform to assess for early evidence of biological activity. Prior neoadjuvant studies have measured drugs targeting the androgen receptor axis, chemotherapeutic agents as well as more novel targets, such as vascular endothelial growth factor receptor, clusterin and platelet derived growth factor.^18^ Assessment of biological activity can include pathological response rates, measures of cell turnover (e.g. Ki67) and apoptosis (e.g. cleaved caspase 3). Changes in gene and/or protein expression specific to the drug target can also be informative. Multiple strategies to restore wild type p53 function are currently in development.^3^ These strategies include agents to normalise p53 folding and structure (e.g. PRIMA-1), agents to reduce p53 degradation and p53 mutant targeted immunotherapy. Drugs to inhibit DNA damage repair kinases aimed at exploiting the already deranged DNA damage repair process also hold promise.^3^ Many of these agents have been tested in preclinical PC models^19^ and entered early clinical testing in other malignancies.^20^

p53 status may also have a role in stratifying patients for more intensive therapy after radical prostatectomy. In two studies of radiation and androgen deprivation therapy for locally advanced PC, evidence of p53 alteration based on immunohistochemistry was associated with MR and PC-specific death.^8,9^ The relationship between p53 and androgen deprivation therapy was conflicting, suggesting a lack of benefit from androgen deprivation therapy in the p53 mutated group in one study, while the other suggested androgen deprivation therapy was imperative in this patient subset. Clearly, more work is required to tease out these relationships.

This study was based on immunohistochemical analysis of whole tissue sections following radical prostatectomy. Work from our group and others has shown that assessing p53 mutations on IHC from tissue microarrays (TMA) has not delivered the same results.^11^ While TMA analysis is convenient and with appropriate sampling technique is suitable for most prostate cancer biomarkers,^21^ our study has shown that p53 is an unusual biomarker that requires analysis of whole tissue sections and may be dismissed by the TMA method. The prognostic significance of the ‘clustering’ of p53-positive tumour nuclei is particularly relevant to the method of analysis. The heterogeneity of p53 staining in prostate cancer tissue has long been recognised.^22^ ‘Clustering’ of p-53-positive tumour nuclei may represent a focal event in one cancer cell that on replication causes a cluster of cells with abnormal p53 protein. Recognising this pattern emphasises the need to examine all regions of disease as inadequate sampling may miss focal regions of p53 immunostaining and lead to misleading results.

This study has confirmed that p53 nuclear accumulation predicts for increased risk of MR and PC-specific mortality, suggesting that p53 mutation is an early lethal aberration in PC progression. Ongoing efforts to target this pathway are warranted.

## Supplementary information

Supplementary file

## Data Availability

All the data analysed supporting the study results can be found at the Garvan Institute for Medical Research, Sydney Australia.

## References

[1] Siegel R, Ma J, Zou Z, Jemal A (2014). Cancer statistics, 2014. CA: a cancer J. Clin..

[2] Kandoth C, McLellan MD, Vandin F, Ye K, Niu B, Lu C (2013). Mutational landscape and significance across 12 major cancer types. Nature.

[3] Kastenhuber ER, Lowe SW (2017). Putting p53 in context. Cell.

[4] Finlay CA, Hinds PW, Tan TH, Eliyahn D, Oren M, Levine AJ (1988). Activating mutations for transformation by p53 produce a gene product that forms an *hsc70-p53* complex withan altered half-life. Mol. Cell Biol..

[5] Guedes LB, Almutairi F, Haffner MC, Rajoria G, Liu Z, Klimek S (2017). Analytic, preanalytic, and clinical validation of p53 IHC for detection of TP53 missense mutation in prostate cancer. Clin. Cancer Res..

[6] Navone NM, Troncoso P, Pisters LL, Goodrow TL, Palmer JL, Nichols WW (1993). p53 protein accumulation and gene mutation in the progression of human prostate carcinoma. J. Natl. Cancer Inst..

[7] Robinson D, Van Allen EM, Wu YM, Schultz N, Lonigro RJ, Mosquera JM (2015). Integrative clinical genomics of advanced prostate cancer. Cell.

[8] Che M, DeSilvio M, Pollack A, Grignon DJ, Venkatesan VM, Hanks GE (2007). Prognostic value of abnormal p53 expression in locally advanced prostate cancer treated with androgen deprivation and radiotherapy: a study based on RTOG 9202. Int J. Radiat. Oncol. Biol. Phys..

[9] Grignon DJ, Caplan R, Sarkar FH, Lawton CA, Hammond EH, Pilepich MV (1997). p53 status and prognosis of locally advanced prostatic adenocarcinoma: a study based on RTOG 8610. J. Natl. Cancer Inst..

[10] Xie W, Regan MM, Buyse M, Halabi S, Kantoff PW, Sartor O (2017). Metastasis-free survival is a strong surrogate of overall survival in localized prostate cancer. J. Clin. Oncol..

[11] Zhang AY, Chiam K, Haupt Y, Fox S, Birch S, Tilley W (2019). An analysis of a multiple biomarker panel to better predict prostate cancer metastasis after radical prostatectomy. Int J. Cancer.

[12] Quinn D. I., Henshall S. M., Head D. R., Golovsky D., Wilson J. D., Brenner P. C., et al. Prognostic significance of p53 nuclear accumulation in localized prostate cancer treated with radical prostatectomy. *Cancer Res*. **60**, 1585–1594 (2000) 10749127

[13] Epstein JI, Egevad L, Amin MB, Delahunt B, Srigley JR, Humphrey PA (2016). The2014 International Society of Urological Pathology (ISUP) Consensus Conference on Gleason Grading of Prostatic Carcinoma: definition of grading patterns and proposal for a new grading system. Am. J. Surg. Pathol..

[14] Carroll AG, Voeller HJ, Sugars L, Gelmann EP (1993). p53 oncogene mutations in three human prostate cancer cell lines. Prostate.

[15] Yang G, Stapleton AMF, Wheeler TM, Truong LD, Timme TL, Scardino PT (1996). Clustered p53 immunostaining: a novel pattern associated with prostate cancer progression. Clin. Cancer Res..

[16] Stapleton AM, Timme TL, Gousse AE, Li QF, Tobon AA, Kattan MW (1997). Primary human prostate cancer cells harboring p53 mutations are clonally expanded in metastases. Clin. Cancer Res..

[17] Kastan MB, Onyekwere O, Sidransky D, Vogelstein B, Craig RW (1991). Participation of p53 protein in the cellular response to DNA damage. Cancer Res..

[18] McKay RR, Choueiri TK, Taplin ME (2013). Rationale for and review of neoadjuvant therapy prior to radical prostatectomy for patients with high-risk prostate cancer. Drugs.

[19] Wang H, Yu D, Agrawal S, Zhang R (2003). Experimental therapy of human prostate cancer by inhibiting MDM2 expression with novel mixed-backbone antisense oligonucleotides: in vitro and in vivo activities and mechanisms. Prostate.

[20] Andreeff M, Kelly KR, Yee K, Assouline S, Strair R, Popplewell L (2016). Results of the phase I trial of RG7112, a small-molecule MDM2 antagonist in leukemia. Clin. Cancer Res..

[21] Rubin MA, Dunn R, Strawderman M, Pienta KJ (2002). Tissue microarray sampling strategy for prostate cancer biomarker analysis. Am. J. Surg. Pathol..

[22] Van Veldhuizen PJ, Sadasivan R, Cherian R, Dwyer T, Stephens RL (1993). p53 expression in incidental prostatic cancer. Am. J. Med Sci..

