# Supplementary material for: p53 nuclear accumulation as an early indicator of lethal prostate cancer
Source: Br J Cancer. 2019 Aug 14;121(7):578–83. doi: 10.1038/s41416-019-0549-8 (PMC6889144; doi:10.1038/s41416-019-0549-8)
Supplement: Supplementary file 1 — Supplementary file [file 41416_2019_549_MOESM1_ESM.pdf]

Supplementary Data Legend

Supplementary Fig 1 Representative photomicrographs demonstrating p53 nuclear accumulation in prostate cancer. A. Cluster positive case - Gleason 4 prostate cancer acinar formation with >12 cells demonstrating p53 nuclear accumulation (brown) (400x magnification). B. Prostate cancer Gleason single grade 4 with no nuclei demonstrating p53 nuclear accumulation (200x). C. Prostate cancer Gleason single grade 4 with >70% of nuclei demonstrating p53 nuclear accumulation (200x).

Supplementary Table 1 Correlations between p53 nuclear staining, histopathological grade, stage and baseline PSA.

Supplementary Table 2 Multivariable analysis of p53 nuclear staining (≥10% p53 positive nuclei) and established baseline prognostic variables and their association with biochemical relapse, clinical relapse and prostate cancer specific survival.

Supplementary Figure 1

Representative photomicrographs demonstrating p53 nuclear accumulation in prostate cancer.

A. Cluster positive case - Gleason 4 prostate cancer acinar formation with >12 cells demonstrating p53 nuclear accumulation (brown) (400x magnification).

B. Prostate cancer Gleason single grade 4 with no nuclei demonstrating p53 nuclear accumulation (200x).

C. Prostate cancer Gleason single grade 4 with >70% of nuclei demonstrating p53 nuclear accumulation (200x).

A

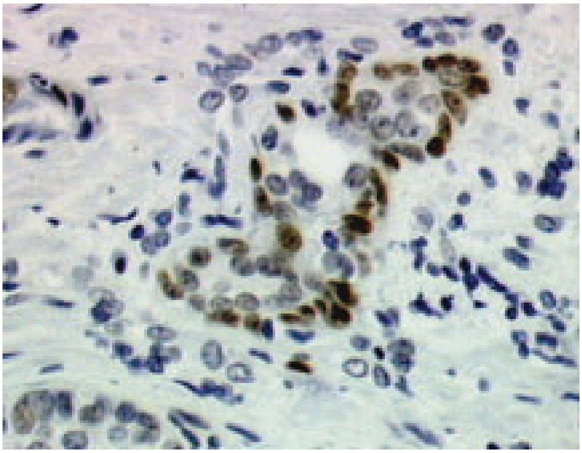

B

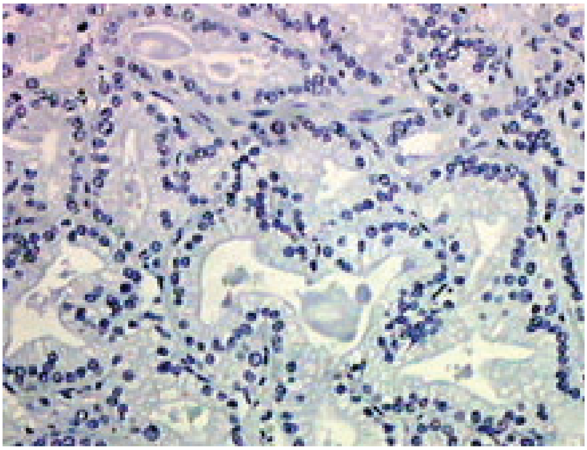

C

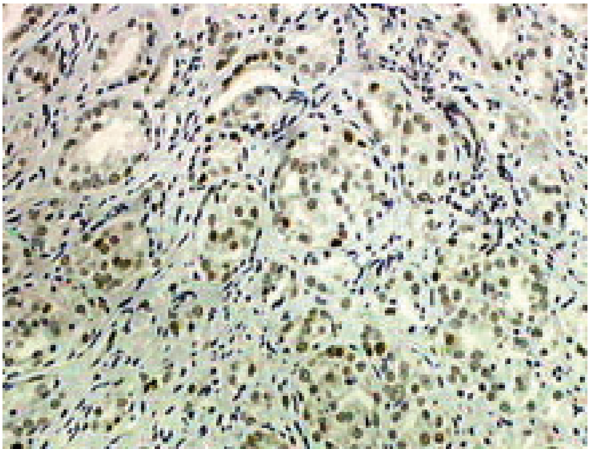

Supplementary Table 1

	N	p53 accumulation strata (% of patients within each group)							p53 cluster	
		0	0-<2% cluster -ve	0-<2% cluster +ve	≥2-<5%	≥5-<20%	≥20%	p	% positive	p
**All**	271	22.5	27.7	12.2	14.4	11.1	12.2		49.8	
**ISUP grade group**	241	23.3	28.7	12.5	13.8	9.6	12.1		47.7	
1	49	32.7	44.9	10.2	4.1	8.2	0		22.4	
2	108	28	28	12.1	14	8.4	9.3		43.5	
3	47	17	17	17	17	12.8	19.1	0.001	66	<0.001
4	12	0	25	8.3	41.7	8.3	16.7		75	
5	25	8	24	12	12	12	32		68	
**Path stage**	270	22.2	27.8	12.2	14.4	11.1	12.2		49.8	
T2N0	127	28.3	33.9	12.6	11.8	6.3	7.1		37.5	
T3AN0	87	20.7	26.4	10.3	14.9	16.1	11.5	<0.001	52.9	<0.001
T3BN0	45	8.9	20	15.6	24.4	11.1	20		71.1	
T4N0	5	40	0	20	0	20	20		60	
TxN+	6	0	0	0	0	33.3	66.7		100	
**preRP PSA**	251	22.7	27.1	12.4	14.7	11.2	12			
<4	12	41.7	16.7	0	16.7	0	25		41.7	
4-10	103	28.2	39.8	7.8	6.8	10.7	6.8	<0.001	32	<0.001
10.1-20	77	19.5	22.1	18.2	14.3	15.6	10.4		57.7	
>20	59	13.6	13.6	15.3	28.8	8.5	20.3		72.9	

Supplementary Table 2

	HR (95% CI), p-value		
**Variable**	*BCR*	*MR*	*PCSM*
**p53 positive tumour nuclei** *≥ 10% vs <10%*	1.7(1.1-2.6) p=0.02	2.4(1.0-5.4) p=0.04	4.9(1.7-15) p=0.004
**Lymph node involvement**✢ *Present vs absent*	p=0.4	10.2(2.2-48) p=0.03	p=0.6
**ISUP grade group**			
1	1	1	1
2	1.7(1.1-2.8) p=0.03	p=0.9	p=0.9
3	2.3(1.3-4.0) p=0.003	p=0.1	p=0.4
4	2.4(1.1-5.2) p=0.07	p=0.053	p=0.9
5	2.0(1.0-4.0) p=0.05	8.6(1.7-44) p=0.009	p=0.09
Baseline PSA[#], ng/ml	p=0.5	p=0.8	p=0.9

Continuous variable, log transformed.
✢ Lymph nodes involved in 6/271 (2%) patients
HR, hazard ratio; CI, confidence interval; BCR, biochemical relapse; MR, metastatic relapse; PCSM, prostate cancer specific mortality; ISUP, International Society of Urological Pathology.
